# Gain of Spontaneous *clpX* Mutations Boosting Motility *via* Adaption to Environments in *Escherichia coli*

**DOI:** 10.3389/fbioe.2021.772397

**Published:** 2021-11-24

**Authors:** Bingyu Li, Chaofan Hou, Xian Ju, Yong Feng, Zhi-Qiang Ye, Yunzhu Xiao, Mingyao Gu, Chunxiang Fu, Chaoliang Wei, Conghui You

**Affiliations:** ^1^ Guangdong Key Laboratory for Genome Stability and Disease Prevention, Health Science Center, Shenzhen University, Shenzhen, China; ^2^ Shenzhen Key Laboratory of Microbial Genetic Engineering, College of Life Sciences and Oceanology, Shenzhen University, Shenzhen, China; ^3^ Shandong Provincial Key Laboratory of Energy Genetics, Key Laboratory of Biofuels, Qingdao Engineering Research Center of Biomass Resources and Environment, Qingdao Institute of Bioenergy and Bioprocess Technology, Chinese Academy of Sciences, Qingdao, China; ^4^ Lab of Computational Chemistry and Drug Design, State Key Laboratory of Chemical Oncogenomics, Peking University Shenzhen Graduate School, Shenzhen, China

**Keywords:** evolvable motility, spontaneous *clpX* mutations, ClpXP protease, protein degradation, motility-related reprogramming of gene expression, stress response, biofilm, *Escherichia coli*

## Abstract

Motility is finely regulated and is crucial to bacterial processes including colonization and biofilm formation. There is a trade-off between motility and growth in bacteria with molecular mechanisms not fully understood. Hypermotile *Escherichia coli* could be isolated by evolving non-motile cells on soft agar plates. Most of the isolates carried mutations located upstream of the *flhDC* promoter region, which upregulate the transcriptional expression of the master regulator of the flagellum biosynthesis, FlhDC. Here, we identified that spontaneous mutations in *clpX* boosted the motility of *E. coli* largely, inducing several folds of changes in swimming speed. Among the mutations identified, we further elucidated the molecular mechanism underlying the ClpX^V78F^ mutation on the regulation of *E. coli* motility. We found that the V78F mutation affected ATP binding to ClpX, resulting in the inability of the mutated ClpXP protease to degrade FlhD as indicated by both structure modeling and *in vitro* protein degradation assays. Moreover, our proteomic data indicated that the ClpX^V78F^ mutation elevated the stability of known ClpXP targets to various degrees with FlhD as one of the most affected. In addition, the specific tag at the C-terminus of FlhD being recognized for ClpXP degradation was identified. Finally, our transcriptome data characterized that the enhanced expression of the motility genes in the ClpX^V78F^ mutations was intrinsically accompanied by the reduced expression of stress resistance genes relating to the reduced fitness of the hypermotile strains. A similar pattern was observed for previously isolated hypermotile *E. coli* strains showing high expression of *flhDC* at the transcriptional level. Hence, *clpX* appears to be a hot locus comparable to the upstream of the *flhDC* promoter region evolved to boost bacterial motility, and our finding provides insight into the reduced fitness of the hypermotile bacteria.

## Introduction

Bacterial motility is one of the most extensively studied biological processes. Flagellar motility helps bacteria to reach favorable environments (Fenchel, 2002), and bacterial flagella also play a crucial role in biofilm formation (Guttenplan and Kearns, 2013) and colonization (Ageorges et al., 2020). Flagellum is a complex organelle, and more than 50 genes are required for flagellar biosynthesis in *Escherichia coli* and *Salmonella typhimurium*. The biosynthesis and operation of flagella are energy-consuming processes, costing 2% of the biosynthetic energy in *E. coli* (Soutourina and Bertin, 2003; Fontaine et al., 2008). Highly motile cells of *E. coli* were observed to show reduced biomass (Wang and Wood, 2011) or growth fitness (Ni et al., 2017) when compared to weakly motile cells. Thus, there is a trade-off between motility and growth, and bacteria need to coordinate flagellum biosynthesis well with cellular growth. As a result, both the synthesis and the function of flagella are finely tuned.

Flagellum biosynthesis is regulated by a hierarchy system in enterobacteria (Soutourina and Bertin, 2003). On the top of the hierarchy, there is only the Class I master regulator, FlhDC. FlhDC activates the expression of all the other flagellar genes belonging to Class II and Class III of the entire flagellar cascade in a direct or indirect mode. Among the Class II genes, *fliA* encodes the sigma 28 (sigma F) factor which activates the expression of flagellar genes in Class III (Fitzgerald et al., 2014). *E. coli* applies many transcriptional factors to tightly control the expression of the *flhDC* operon, including Crp, NtrC, CysB, HNS, and LrhA (Keseler et al., 2017). At the posttranslational level, FlhDC is degraded by the ClpXP ATP-dependent protease in *S. typhimurium* (Tomoyasu et al., 2003) and enterohaemorrhagic *E. coli* (Kitagawa et al., 2011) as the FlhDC complex was accumulated when ClpX was deleted in these species. Studies *in vitro* demonstrated that ClpXP could directly recognize and degrade the FlhDC complex, but the tag sequence of FlhDC recognized by ClpXP remains obscure (Takaya et al., 2012).

Intriguingly, bacterial motility shows a very strong evolvable feature depending on the environment. Evolutionary experiments demonstrated that hypermotile *E. coli* could be isolated from cultures of non-motile *E. coli* grown on soft agar plates (Barker et al., 2004; Wang and Wood, 2011; Lee and Park, 2013; Fahrner and Berg, 2015; Zhang et al., 2017) or grown in resting liquid culture without shaking (Parker et al., 2019). Most of these hypermotile isolates carried transposition of insertion sequence (IS) elements to the upstream of the *flhDC* promoter region, which upregulates the expression of this operon and the entire flagellar cascade greatly. Alternatively, some of the isolates showed increased expression of *flhDC* by carrying mutation in LrhA (Lee and Park, 2013; Parker et al., 2019), a repressor of *flhDC* (Lehnen et al., 2002). Thus, evolved mutations increased transcriptional expression of FlhDC and enhanced bacterial motility largely. Recently, several evolutionary studies starting with motile *E. coli* strains identified mutants carrying single-point mutations in ClpX during the process of evolution (Fraebel et al., 2017; Liu et al., 2019). One reported an evolved mutant carrying ∼30% higher motility than the parent strain (Fraebel et al., 2017), whereas the evolved mutant in other reports did not show significantly enhanced motility (Liu et al., 2019). Therefore, evolved mutations controlling FlhDC degradation by ClpXP at the posttranslational level also enhanced bacterial motility, but slightly.

In this work, we found that the spontaneous mutations in *clpX* could boost bacterial motility largely, making *clpX* a hot locus comparable to the upstream of the *flhDC* promoter region during the evolution of bacterial motility. We further revealed the molecular mechanism of the identified ClpX mutants that mediated FlhDC accumulation, characterized the degradation signal of FlhDC recognized by ClpXP, and reasoned the trade-off between motility and growth fitness at the molecular level.

## Results

### Phenotypes of Hypermotile *E. coli* Identified

**FIGURE 1:**
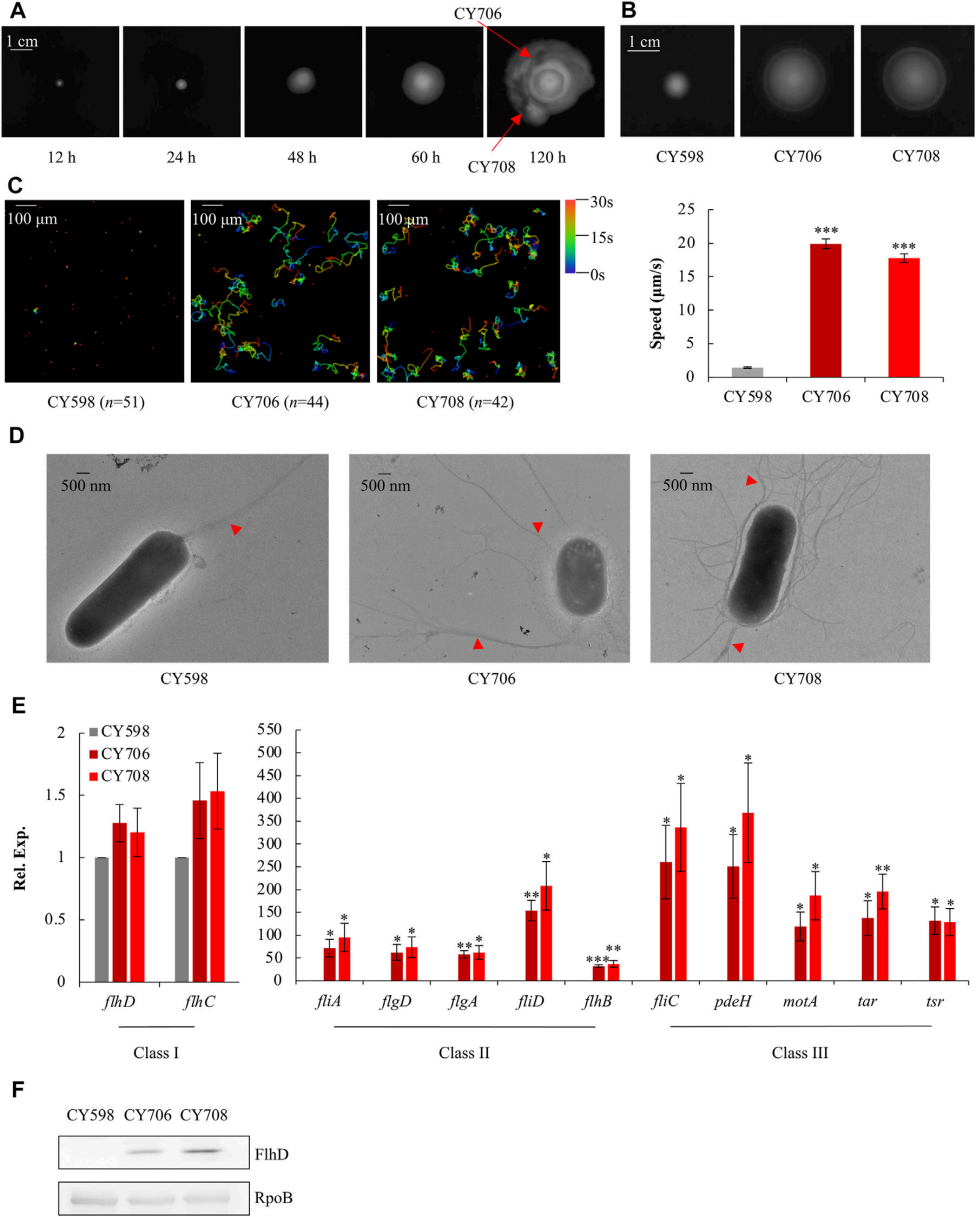
Isolation and characterization of hypermotile mutants evolved from CY598. **(A)** The swimming zones formed by CY598 on the soft agar plate (0.4%) at different time points at 37°C. Putative hypermotile mutants were isolated from arrowed areas. **(B)** Swimming motility of the three strains on soft agar plates (0.25%) after 6-h incubation at 37°C. Three independent cultures were tested, and one representative image is shown. **(C)** Swimming speed of the three strains in liquid culture. The left plots showed the trajectories of the single-cell movement of the three strains. The rainbow colors correspond to the tracking time, and “*n*” represents the number of the cells tracked in the image. The swimming speed was expressed as the average ±S.E.M. in the right plots, calculated from 128 to 141 individual trajectories combined from three independent experiments. ***p ≤ 0.001 *versus* the swimming speed of the wild-type strain, CY598. **(D)** Flagella production of single cells of the three strains. One representative image of at least three independent cultures is shown. Red arrows mark representative flagella. **(E)** The expression of example genes selected from Class I, Class II, and Class III flagella genes in the three strains detected by qRT-PCR. The mRNA level of each gene in CY598 was normalized to 1, and that CY706 and CY708 were determined relative to this value. The relative expression was shown as the average ±S.E.M. of three independent experiments. *p ≤ 0.05, **p ≤ 0.01, ***p ≤ 0.001 *versus* the relative expression by the wild-type strain, CY598. **(F)** The protein levels of FlhD in the three strains. RpoB was used as a loading control. Three independent western blots were tested, and one representative result is shown.

The *E. coli* K-12 wild-type strain NCM3722 is a non-motile strain (Cremer et al., 2019) (Supplementary Figure S1) since it carries a lone missense mutation (N87K) in the gene of *fliC* which encodes flagellin (Lyons et al., 2011), possibly affecting the function of its flagella. Thus, this mutated *fliC* of NCM3722 was complemented to the wild-type *fliC* of another widely used *E. coli* K-12 wild-type strain MG1655, resulting in the parent strain, CY598. With the complemented FliC, CY598 was weakly motile on the soft agar plate (Supplementary Figure S1). In order to obtain hypermotile mutants, CY598 was inoculated into a soft agar plate, and the appearance of swimming zones was recorded at different time points (Figure 1A). After incubation for 120 h, big swimming zones appeared (Figure 1A). Two strains (CY706 and CY708) were isolated from different areas of the swimming zones, and their motility was tested. When compared to the parent CY598, they showed 2–3 folds higher swimming motility in soft agar plates (Figure 1B) and approximately 10-fold faster swimming speed under the microscope (Figure 1C, Supplementary Videos S1–S3). The flagella produced by these strains were further observed under the transmission electron microscope (TEM) (Figure 1D). CY706 and CY708 produce a much larger number of flagella than the parent CY598, supporting the hypermotility of the two strains. Then, we investigated the expression level of genes involved in motility by quantitative real-time PCR (qRT-PCR). The expression of several example genes in Class I, II, and III of the entire flagellar cascade was tested (Figure 1E). The genes in Class II and III showed >30 folds increased expression in CY706 and CY708 as compared to that in CY598. However, there were no significant expression changes of *flhDC*, encoding the master regulator of motility regulon in Class I. Consequently, we assessed the protein amount of FlhD by western blots and found that the FlhD protein was highly accumulated in both CY706 and CY708 (Figure 1F). Thus, the accumulation of FlhD protein in these evolved strains might mediate the hypermotile phenotype observed.

### ClpX^V78F^ Induced High Accumulation of FlhDC in the Hypermotile Strain

**FIGURE 2:**
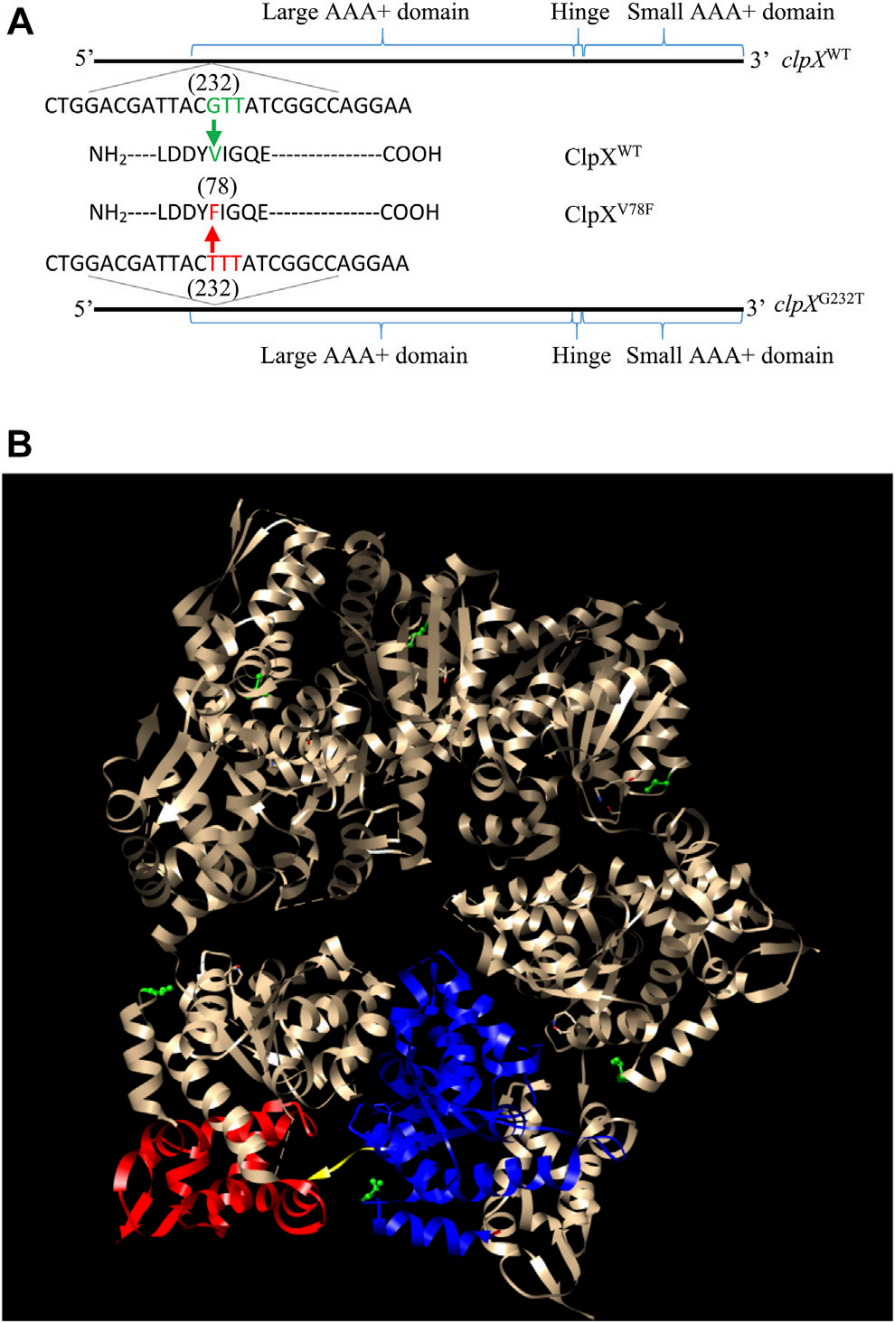
Identification of the ClpX^V78F^ mutation in the hypermotile isolates of CY706 and CY708. **(A)** The DNA and amino acid sequence of the *clpX* variant identified. The corresponding positions of the large AAA+ domain, small AAA+ domain, and the hinge of the ClpX protein in its DNA sequence are marked. The G232T mutation in the *clpX* gene changed the codon of valine (GUU) into the codon of phenylalanine (UUU), resulting in ClpX^V78F^. **(B)** The structure of a ClpX hexamer (PDB code 3HTE) (Glynn et al., 2009). The 78th valine (V78) in Chain A-F has been highlighted in green (ball and stick). Chain B of the hexamer was used for showing the large AAA+ domain (blue ribbon), small AAA+ domain (red ribbon), and the hinge (yellow region). The highlighting of domains and residues was performed using the software Chimera 1.14 (Pettersen et al., 2004).

To find out the possible reasons that induced the accumulation of FlhD in CY706 and CY708, we performed whole-genome sequencing of CY598, CY706, and CY708. As compared to the genome of CY598, we found that CY706 and CY708 shared one common point mutation. This common G232T mutation located at the 232nd nucleotide in the *clpX* gene, which leads to the change of amino acid in the ClpX protein from valine to phenylalanine at position 78, giving the mutated ClpX^V78F^ (Figure 2A). This V78F mutation was located in the large AAA+ domain of ClpX (Stinson et al., 2013) (Figure 2B). The shared ClpX^V78F^ mutation might explain the highly motile phenotype mediated by the accumulation of FlhD that we observed both in CY706 and CY708.

**FIGURE 3:**
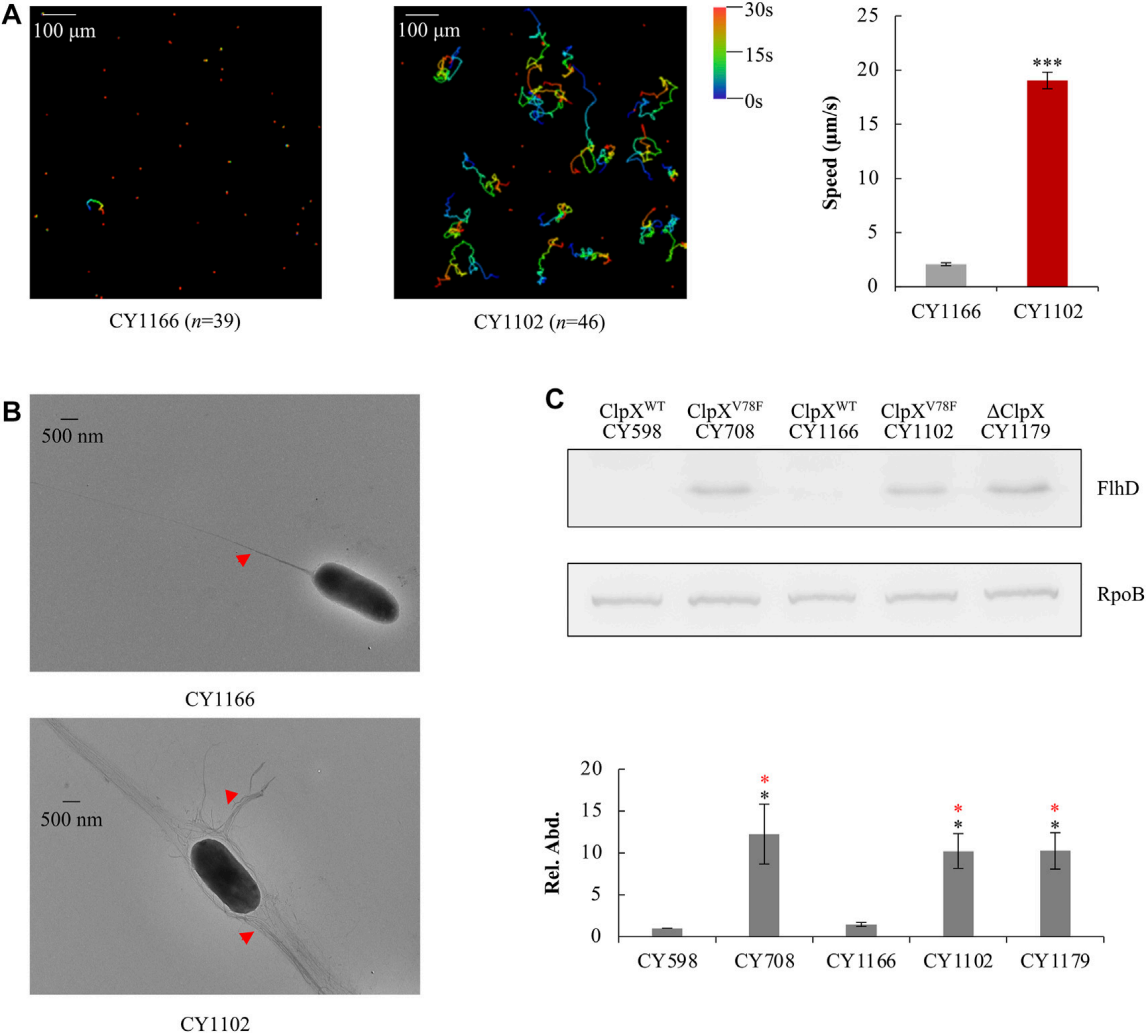
Effect of ClpX^V78F^ on FlhD accumulation in the hypermotile mutants. **(A)** Swimming speed of the two strains in liquid culture. The left plots showed the trajectories of the single-cell movement of the two strains. The rainbow colors and “*n*” were indicated as shown in Figure 1C. The swimming speed was expressed as the average ±S.E.M. in the right plots, calculated from 135 to 165 individual trajectories combined from three independent experiments. ***p ≤ 0.001 *versus* the swimming speed by CY1166. **(B)** Flagella production of single cells of the two strains. Image was shown as that in Figure 1E. **(C)** The protein levels of FlhD in the five strains. RpoB was used as a loading control. One representative result of the western blots is shown in the upper plot. The protein level of FlhD in CY598 was normalized to 1, and that in other strains was determined relative to this value. The relative protein level (Rel. Abd.) is shown as the average ±S.E.M. of the three independent experiments in the lower plot. *p ≤ 0.05 *versus* the relative protein level in CY598 (black) or CY1166 (red).

To verify this point, using the λ Red system (Datsenko and Wanner, 2000), we replaced the wild-type *clpX* gene in the parent strain CY598 by the mutated *clpX* (G232T), resulting in the strain CY1102, and complemented the mutated *clpX* (G232T) in the hypermotile strain of CY708 with the wild-type *clpX* gene in parallel, resulting in the strain CY1166. Then, their motility, flagella, and the amount of FlhD protein were individually assayed. Similar to CY708 and CY706 (Figures 1B,C), CY1102 carrying ClpX^V78F^ was highly motile (Figure 3A, Supplementary Video S4) and produced larger number of flagella (Figure 3B). In contrast, CY1166 carrying wild-type ClpX was weakly motile (Figure 3A, Supplementary Video S5) and produced less flagella (Figure 3B), exhibiting a similar phenotype as the parent CY598 (Figures 1B,C). In consistence with these observations, FlhD was accumulated highly in CY1102 to a similar level as that observed in CY708, but no accumulation of FlhD was observed in CY1166 (Figure 3C). At the same time, FlhC of the FlhDC regulator was also identified to be highly accumulated in the ClpX^V78F^ background (Supplementary Figure S2). These evidences suggested that it was the mutated ClpX^V78F^ that mediated the accumulation of FlhDC and enhanced the motility. Moreover, we monitored the protein level of FlhD in the ClpX null background because the FlhDC complex was highly accumulated in the absence of the ClpXP protease (Tomoyasu et al., 2003; Kitagawa et al., 2011). Interestingly, the amount of FlhD was increased to a similar level in the ClpX null background as that observed in the ClpX^V78F^ background (Figure 3C). Thus, the V78F mutation in ClpX seems to inactivate the ability of the ClpXP to hydrolyze FlhD completely. All lines of evidences suggested that the *clpX* ^G232T^ mutation contributed to the hypermotile phenotype observed.

### Identification of More Hypermotile Mutants Carrying Spontaneous Mutations in the *clpX* Gene

People had reported that single-point mutations in *clpX* could enhance the motility in *E. coli* (Fraebel et al., 2017; Liu et al., 2019)*.* Although the reported influence of these mutations on bacterial motility was much less than the enhancement induced by the ClpX^V78F^ mutation identified here, these observations indicated that the gene of *clpX* could be a hot locus to boost bacterial motility.

To further explore this point, applying the same method (Figure 1A), we performed a second round of selection for the hypermotile mutants. Intriguingly, more isolates carrying different point mutations in the *clpX* gene were characterized. Out of the total 18 hypermotile strains we isolated, four mutants carried the same Q289* (C865T) mutation (* indicates stop codon) and one mutant carried an L317R (T950G) mutation (Supplementary Figure S3). Thus, these evidences suggested that the gene of *clpX* could be a hot locus mutated spontaneously to boost bacterial motility.

How would these mutants induce the hypermotile phenotype? In the case of the ClpX carrying the Q289* mutation (Supplementary Figure S3), a truncated ClpX without the hinge and the small AAA+ domain would be generated (Stinson et al., 2013), which would lose its ability to degraded FlhDC. In the case of the ClpX carrying L317R mutation, the residue of L317R was localized in the hinge domain of ClpX (Supplementary Figure S3) (Glynn et al., 2012). Since leucine and arginine show distinct biochemical features with one hydrophilic and the other hydrophobic, the L317R mutation would affect the protease function of ClpX by changing the hinge structure. However, in the case of the ClpX carrying V78F mutation, it was not straightforward to predict the effect that this mutation would bring to the function of the mutated ClpX. The residue of V78F was located in the large AAA+ domain of ClpX (Stinson et al., 2013) (Figure 2). Since both valine and phenylalanine are hydrophobic amino acids except that they carry different sizes of side chains, it is quite a mystery how this V78F mutation would inactivate the ability of the ClpXP to hydrolyze FlhD (Figure 3C). Thus, the following study was to discover the molecular mechanism of ClpX^V78F^ mediated high accumulation of FlhDC.

### Molecular Mechanisms of FlhDC Accumulation Mediated by ClpX^V78F^

**FIGURE 4:**
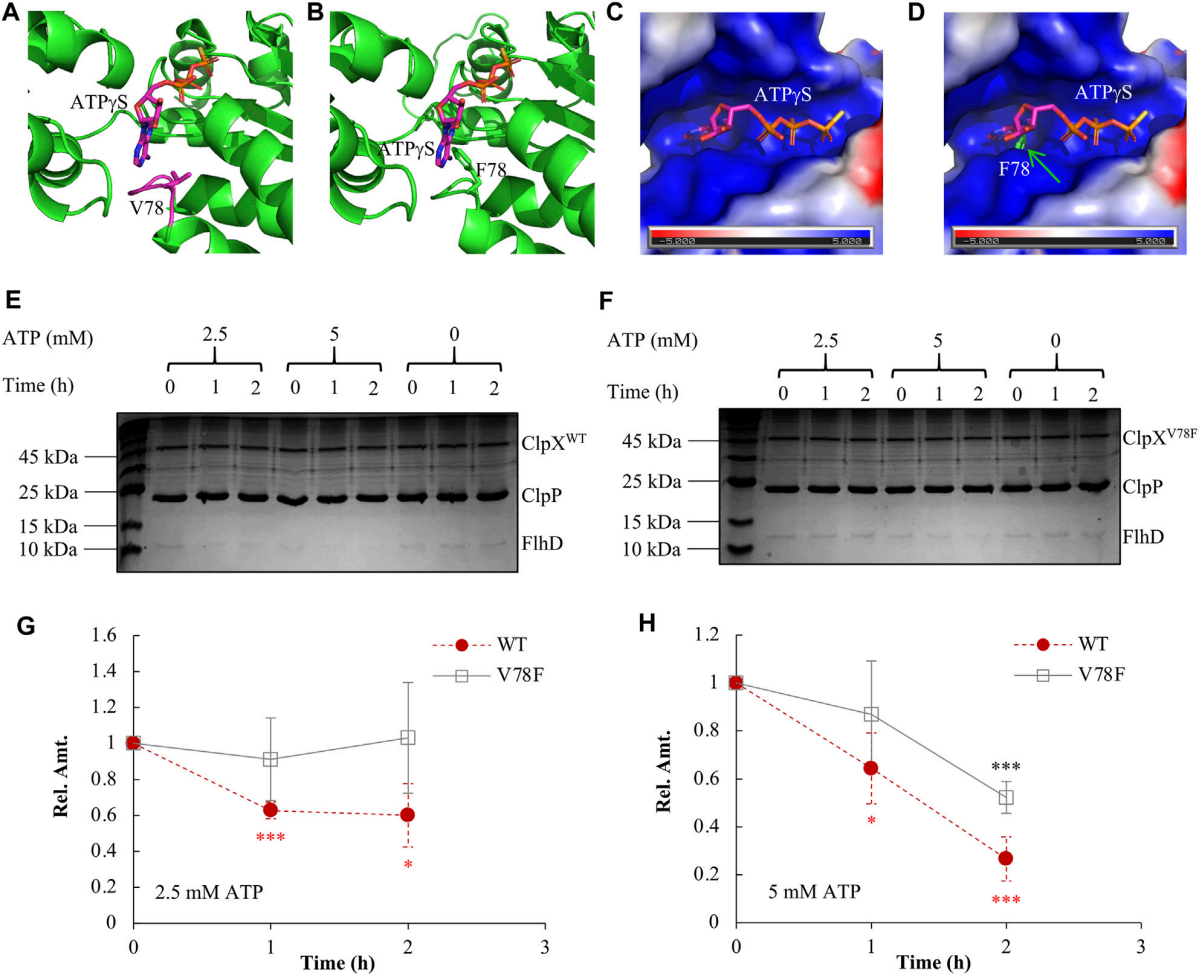
Elucidating the molecular mechanism of FlhDC accumulation mediated by ClpX^V78F^. **(A)** The 3D structure of the wild-type ClpX interacting with ATPγS (PDB code 4I81). **(B)** The modeled 3D structure of the ClpX^V78F^ interacts with ATPγS. In **(A)** and **(B)**, ATPγS and V78/F78 are rendered as sticks while all other parts are shown as cartoon. The interaction mode between ATPγS and the ClpX^V78F^ is modeled by superposing the Cα atoms of ClpX^V78F^ to those of wild-type ClpX (chain A of PDB code 4I81). **(C)** The molecular surface representation of the ATP-binding pocket of wild-type ClpX. The surface is colored according to the electrostatic potential calculated using the APBS tool (Baker et al., 2001; Dolinsky et al., 2004) in PyMOL, where blue and red colors, respectively, indicate positive and negative potential. The electrostatic potential of the binding pocket is mainly positive, which is complementary to the negative charge of ATP especially in the three phosphate groups. **(D)** The molecular surface of the wild-type ClpX superposed with ClpX^V78F^. The F78 of ClpX^V78F^ is rendered as sticks, and it protrudes from the molecular surface of the wild-type ClpX introducing a steric hindrance effect at the binding pocket of ATPγS. **(E)** The *in vitro* degradation assay with ClpX^WT^P for FlhD. As indicated, a proper amount of ClpX^WT^ (2 μM), ClpP (4.3 μM), and FlhD (0.65 μM) was incubated together at the presence of 0, 2.5, or 5 mM ATP. The reaction mix obtained at time 0, 1, and 2 h was checked using SDS-PAGE electrophoresis followed by Coomassie-brilliant-blue staining. The shown result is a representative of three independent experiments. **(F)** The *in vitro* degradation assay with ClpX^V78F^P for FlhD. The assay was performed as described above in Figure 4E, except using ClpX^V78F^ (2 μM) instead of ClpX^WT^ (2 μM). The shown result is a representative of three independent experiments. **(G–H)** FlhD quantification during the process of *in vitro* degradation at the presence of 2.5 mM **(G)** or 5 mM **(H)** ATP. The protein bands of FlhD in panels **(E)** and **(F)** were quantified using the software ImageJ. The amount of FlhD at time 0 h was set as 1, and that at other time points was determined relative to this value. FlhD degradation at the presence of 0 mM ATP was used as a control for normalization. The FlhD relative amount (Rel. Amt.) is shown as the average ±S.E.M. of the three independent experiments. *p ≤ 0.1, ***p ≤ 0.01 *versus* the FlhD relative amount at time 0 h in the reaction with ClpX^WT^ (red) or that in the reaction with ClpX^V78F^ (black).

We had shown that the FlhD in the ClpX^V78F^ background was accumulated to a similar level as that in the ClpX null background (Figure 3C). What could be the reasons? First, one possibility is that the protein of ClpX^V78F^ is unstable so that it makes strains carrying ClpX^V78F^ as a ClpX null strain. This hypothesis was ruled out by the observation that the protein level of the two types of ClpX proteins showed no significant difference between the ClpX^V78F^ mutants and the strains carrying the wild-type ClpX (Supplementary Figure S4). Next, ClpXP is an ATP-dependent protease (Fei et al., 2020). The structure study indicated that ATP bound to ClpX *via* contacting the side chains of V78 and I79 (Stinson et al., 2013). When both V78 and I79 were mutated into A with a very smaller side chain, the mutated ClpX required a higher concentration of ATP to support ATP hydrolysis and protein degradation *in vitro* (Stinson et al., 2013). Will the single V78F point mutation carrying a bigger side chain affect the ATP binding to ClpX by itself? To answer this question, using the I-TASSER server (Yang and Zhang, 2015), we modeled the structure of ClpX^V78F^ by specifying chain A of the reported wild-type ClpX structure (PDB code 4I81) as the template. Compared to V78 in the wild-type ClpX (Figure 4A), the F78 in the mutated ClpX was much closer to the bound ATPγS (Figure 4B). The bulky aromatic side chain of F78 protruded into the ATP-binding pocket and occupied a substantial part of the pocket (Figures 4C,D). As a result, it will be difficult for ATP to bind to the ClpX^V78F^ due to the steric hindrance effect produced by the bulky side chain of the F78 residue. Thus, the mutated ClpXP might lose its ability to degrade FlhDC.

We further purified ClpX^WT^ and ClpX^V78F^ and compared their *in vitro* ability of protein degradation using FlhD as the target. We found that the wild-type ClpXP protease degraded FlhD effectively at the presence of either 2.5 millimolars (mM) or 5 mM ATP (Figure 4E, Figures 4G–H). As expected, it showed higher degradation efficiency with a higher concentration of ATP. In contrast, the degradation activity of the mutated ClpXP protease was largely impaired (Figure 4F, Figures 4G–H). No FlhD degradation was observed at 2.5 mM ATP, but a partial activity of FlhD degradation existed at 5 mM ATP, indicating a higher ATP concentration could slightly restore the protease function of the mutated ClpXP. These results indicate that the V78F mutation affected the affinity of ClpX^V78F^ to ATP and, therefore, the activity of the mutated ClpXP protease during degradation of FlhD, which is consistent with the prediction described above using the structure-modeling methods. In other words, ClpX^V78F^ is more sensitive to deprivation of ATP than ClpX^WT^, at least during degradation for certain targets like FlhD. Given that under most environmental conditions, the ATP level within bacterial cells is limited and tightly tuned (Chapman et al., 1971; Sielaff et al., 2018; Meyrat and von Ballmoos, 2019), and a single-point mutation such as ClpX^V78F^ could extensively affect the stability of many proteins and physiological processes.

**FIGURE 5:**
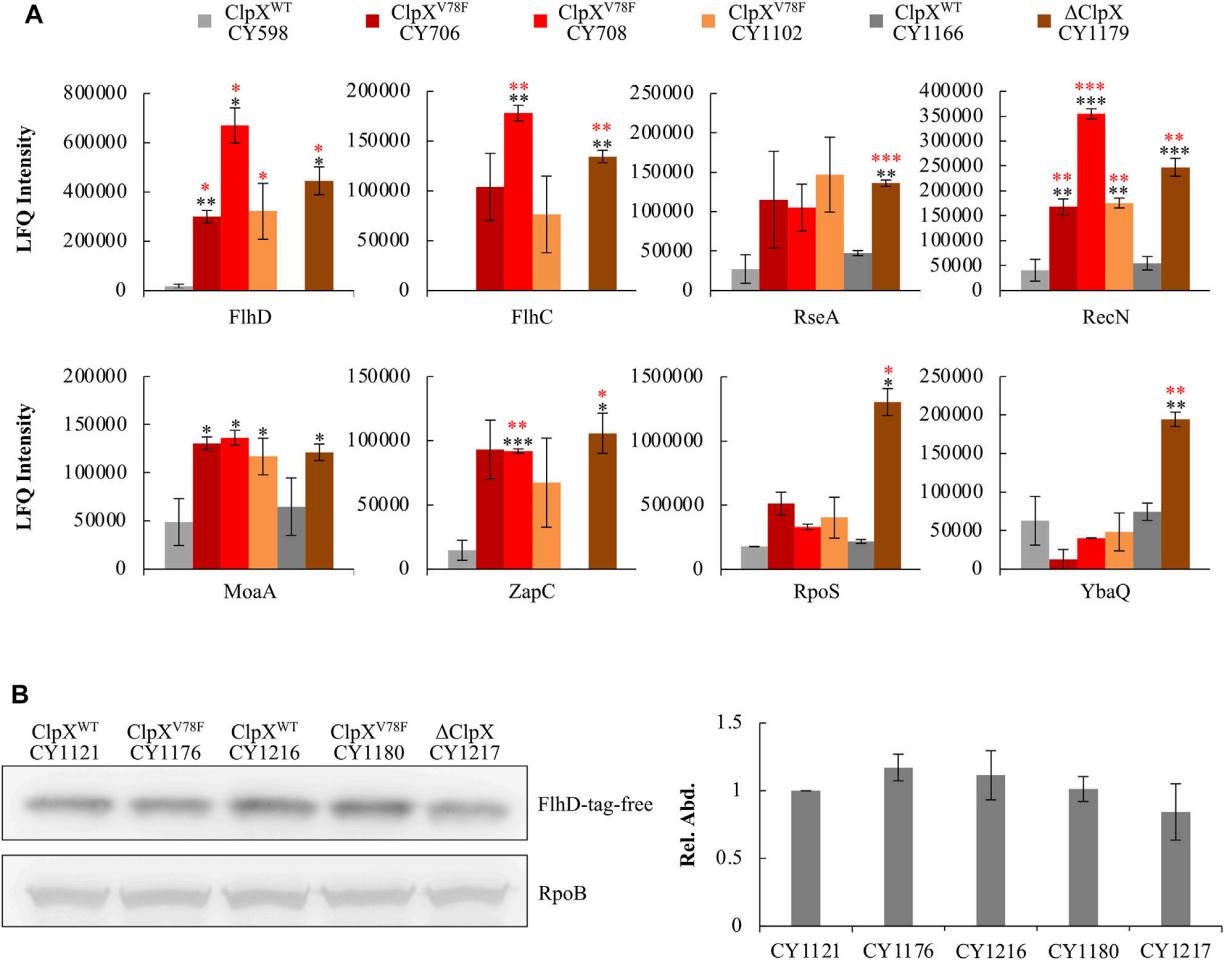
Response of known ClpXP targets to the ClpX^V78F^ mutation and identification of the specific tag of FlhD recognized for ClpXP degradation. **(A)** Proteomic results showing the expression of proteins identified to be degraded by ClpXP protease in the six strains. The data were expressed as average ±S.E.M. of the two to three independent proteomic assays. *p ≤ 0.1, **p ≤ 0.05, ***p ≤ 0.01 *versus* the expression of proteins by CY598 (black) or CY1166 (red). **(B)** The western blot result showing protein levels of the C-terminal tag deleted FlhD (FlhD-tag-free) in strains carrying ClpX^WT^, ClpX^V78F^, or deleted ClpX. Strains harboring the chromosomal FlhD-tag-free were individually derived from CY598 (ClpX^WT^), CY708 (ClpX^V78F^), CY1166 (ClpX^WT^), CY1102 (ClpX^V78F^), and CY1179 (∆ClpX). RpoB was used as a loading control. One representative result of the western blots is shown **(left plot).** The protein level of FlhD-tag-free in CY1121 was normalized to 1, and that in other strains was determined relative to this value. The relative protein level is shown as the average ±S.E.M. of the three independent experiments **(right plot)**.

Moreover, this ClpXP mutant might fail to hydrolyze other protein targets accordingly. To verify this point, we integrated a global proteome study with a transcriptome study to investigate how the other targets of ClpXP changed among the ClpX^V78F^ mutants, and the ClpX null and wild-type strains. A target of ClpXP protease was expected to show changes at the protein level but not at the transcription level. Including FlhD and FlhC, eight targets of ClpXP protease (Flynn et al., 2003; Nagashima et al., 2006) were identified to show significant changes solely at the protein level (Figure 5A). As expected, the FlhD protein accumulated in the strains carrying ClpX^V78F^ to a level comparable to that in the ClpX null strain, which fitted to the previous data of the western blots well (Figure 3C). Interestingly, the other five proteins, including FlhC, RseA, RecN, MoaA, and ZapC, were changed similarly as the FlhD protein among these strains. But two of them (RpoS and YbaQ) were affected differently as their protein amount accumulated more in the ClpX null background when compared to the ClpX^V78F^ background. Given that the bulk of the targets of ClpXP protease identified in this work showed similar changes in the ClpX null background and in the ClpX^V78F^ background, this supported our hypothesis that this point mutation of ClpX^V78F^ inactivated most of its protease activity *via* affecting ATP binding. However, the mutated ClpXP protease could still retain slight residue ability to degrade some targets possibly including RpoS and YbaQ as we identified here, indicating ClpXP protease could have varied specificity to its targets.

### Identification of the Degradation Signal of FlhDC Recognized by ClpXP

Although FlhDC is reported to be degraded by ClpXP, the degradation signal recognized by ClpXP has never been reported. Since FlhC could not be degraded by ClpXP without forming a complex with FlhD (Takaya et al., 2012), FlhD might contain the degradation signal. Since the C-terminal five amino acid residues (RKKRA) of FlhD are highly similar to the reported degradation signals (RKKAI) of the other proteins recognized by ClpXP (Flynn et al., 2003), the C-terminal RKKRA of FlhD could function as the signal tag for ClpXP recognition. Indeed, when we deleted the C-terminal five amino acid residues, the truncated FlhD accumulated in the ClpX wild-type background to a similar level as that observed in the ClpX^V78F^ and ClpX null mutants (Figure 5B). Additionally, the amount of wild-type FlhD accumulated in the ClpX^V78F^ background was comparable to the amount of the truncated FlhD detected in the ClpX^WT^ or ClpX^V78F^ background (Supplementary Figure S5). Therefore, the C-terminal RKKRA of FlhD functioned as the signal tag for ClpXP recognition. As a result, ClpXP directly regulates FlhDC *via* hydrolysis in *E. coli* K-12 strains *in vivo* in a way similar to that it degrades other known protein targets.

### Antagonistic Expression Between Motility Genes and Genes Relating to Biofilm Formation and Stress Response

**FIGURE 6:**
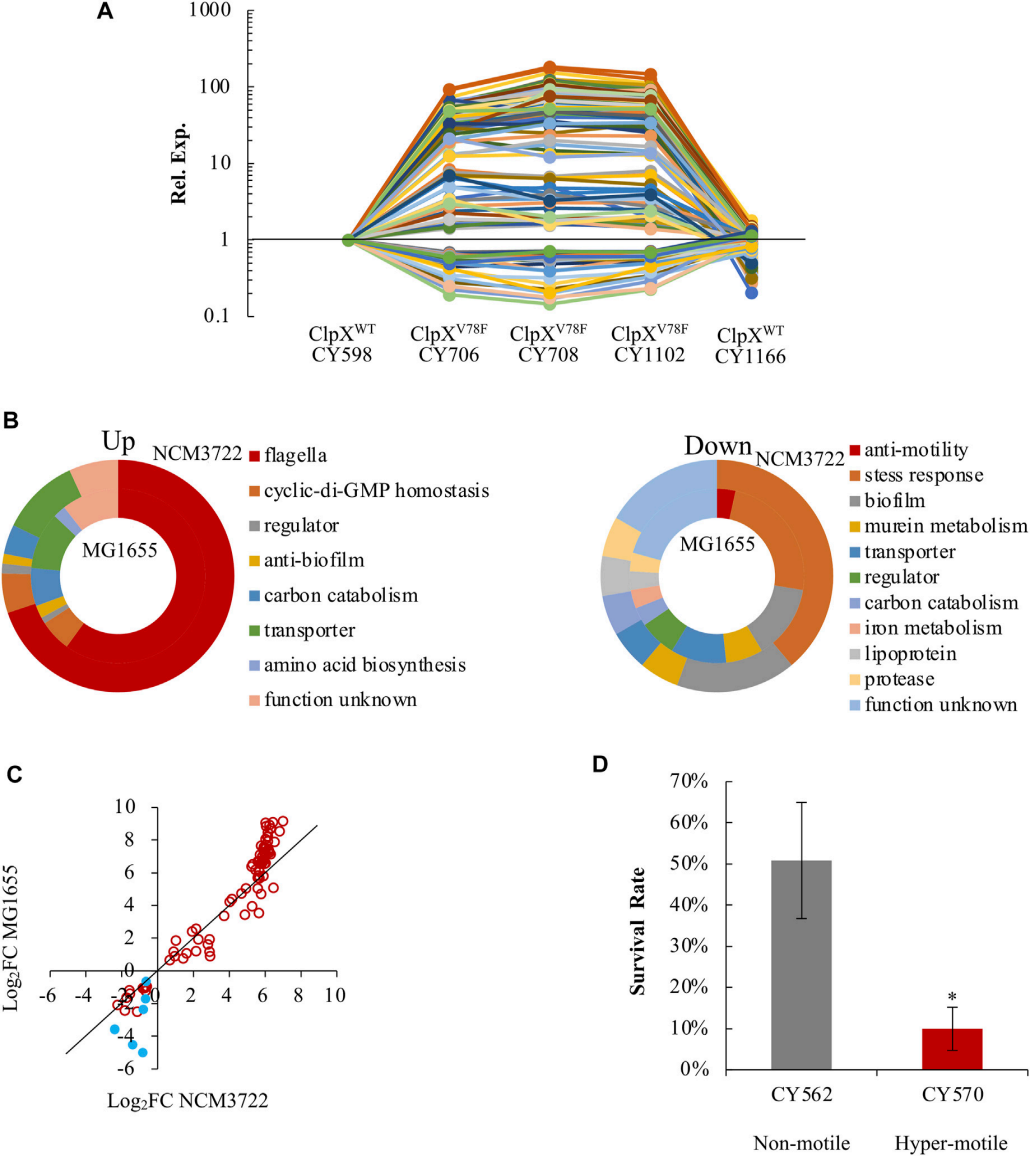
*E. coli* reprograms its global gene expression when changing from non(weak)-motile to hypermotile status. **(A)** The expression trends of the 114 DEGs among the hypermotile strains (CY706, CY708, CY1102) and weak-motile strains (CY598, CY1166). See Supplementary Table S1. The expression of each gene in CY598 was normalized to 1, and that in other strains was determined relative to this value. The relative expression is shown as the average of the three independent RNAseq assays. **(B)** Diagrams grouping the upregulated genes **(upper)** and downregulated genes **(lower)** obtained between the hypermotile strains *versus* the non(weak)-motile strains by the known functions of their gene products. The percentage of the genes’ number in each functional group is shown. See Supplementary Table S1. The results obtained in the NCM3722 and MG1655 background are, respectively, shown in the outer and inner doughnut charts. **(C)** The fold change (FC) of the expression of the 91 genes between the hypermotile and non(weak)-motile strains in NCM3722 background was plotted against that in MG1655 background. The data were shown as Log_2_FC. Genes encoding known stress-response factors were highlighted as blue solid circles. To guide eyes, the black line is shown as y = x. **(D)** The survival rate of non-motile and hypermotile MG1655 strains after 5-min challenge of pH 2.5. The survival rate is shown as the average ±S.E.M. of the three independent experiments. *p ≤ 0.05 *versus* the survival rate of CY598.

Finally, we analyzed the transcriptome of these hypermotile (CY706, CY708, CY1102) and weakly motile strains (CY598, CY1166) systematically. In this process, we could reveal the FlhD or FliA regulons by identifying genes that were expressed similarly as the flagella genes. In parallel, since flagella synthesis is energetically expensive (Soutourina and Bertin, 2003; Fontaine et al., 2008), flagella synthesis in the hypermotile mutant might affect the biosynthesis of other processes and the corresponding genes, and our global transcriptome study could discover those genes. Indeed, we obtained both up- and down-expressed genes in the hypermotile strains. In total, 114 genes were identified as differentially expressed, with 85 genes being upregulated and 29 genes being downregulated in the hypermotile strains (Supplementary Table S1, Figure 6A).

As expected, the bulk of these upregulated genes were reported to be regulated by FlhDC in a direct or indirect mode, including the 51 flagella genes and genes encoding phosphodiesterases which hydrolyze c-di-GMP (Figure 6B). We further identified that four additional genes (*argI*, *lgoT*, *rcsA*, and *yahA*) were also regulated by FlhDC in a direct or an indirect mode since their expression was reduced significantly when FlhDC was deleted (Supplementary Figure S6). We noted that the gene of *yahA* (*pdeL*) encodes a c-di-GMP-specific phosphodiesterase. The FlhDC-regulated PdeH c-di-GMP phosphodiesterase was well-studied which maintains the low c-di-GMP level in order to promote motility (Guttenplan and Kearns, 2013; Serra and Hengge, 2019). The PdeL identified here could be another phosphodiesterase controlled by FlhDC directly or indirectly which works together with PdeH to control the level of c-di-GMP (Reinders et al., 2016).

Interestingly, a bulk of these downregulated genes functioned in biofilm formation, including *bhsA*, *qseBC,* and *ecpB* (Zhang et al., 2007; Garnett et al., 2015; Li et al., 2020), and functioned in stress response resistance, especially acid stress, including *gadW*, *gadX*, *hdeD*, *slp*, *ygiW*, *uspD*, and *iraP* (Nachin et al., 2005; Burton et al., 2010; Krin et al., 2010; Lee et al., 2010; Seo et al., 2015; Dorich et al., 2019) (Supplementary Table S1, Figure 6B). As a result, genes involved in the bacterial motility and genes related to biofilm formation and stress response seemed to show antagonistic expression.

To test if this antagonistic expression is a specific observation or a common phenomenon, we compared the transcriptome of two MG1655 variants (CY570 and CY562), with one hypermotile and the other one non-motile. The hypermotile MG1655 (CY570) carried an IS insertion upstream of the *flhDC* promoter (Supplementary Table S2 of strain list), and *flhDC* was highly expressed in the hypermotile MG1655 strain (Supplementary Figure S7). Approximately 80% of the 114 genes identified in the NCM3722 background were also differentially expressed between the hypermotile and the non-motile MG1655 strains (Supplementary Figure S8). Similarly, genes functioned in motility were upregulated, but genes related to stress resistance and biofilm formation were downregulated in the hypermotile MG1655 strain (Figure 6B, Supplementary Table S1). Thus, the antagonistic expression between motility genes and genes related to biofilm formation and stress response could be a common phenomenon in the K-12 strains of *E. coli*. Since the stress-related genes showed a bigger fold of change in the MG1655 background (Figure 6C), we compared the acid stress response of the non-motile and hypermotile MG1655 strains. Indeed, the hypermotile MG1655 was less resistant to the acid stress than the non-motile strain (Figure 6D).

As a result, in the hypermotile strains, cells reduced the expression of genes related to biofilm formation and stress response in order to reallocate more resources for the biosynthesis of flagella. Given the important role of stress response in bacteria survival (Schellhorn, 2020), these evidences showed molecular reasons for the reduced fitness of the hypermotile bacteria.

## Discussion

In the present study, we reported that adaptive evolution on soft agar could cause spontaneous mutations in *clpX*, which was an efficient way to boost bacterial motility, and supplied molecular reasons underlying the resulted hypermotile bacteria.

The ClpX mutations identified in this work enhanced motility largely. The mutants showed folds of changes in motility (Figures 1B,C), comparable to the effect of IS insertion to the upstream of the *flhDC* promoter (Barker et al., 2004; Wang and Wood, 2011; Lee and Park, 2013; Fahrner and Berg, 2015; Zhang et al., 2017). Recent studies also identified mutants carrying single-point mutations in *clpX* during the process of motility evolution (Fraebel et al., 2017; Liu et al., 2019). However, these evolved mutants enhanced the motility of *E. coli* only slightly. The different effects of the evolved ClpX mutants on the motility observed in the present work and in the literature could depend on the features of strains applied. Our parent strain is a weakly motile *E. coli* K-12 wild-type NCM3722 strain with repaired *fliC* (Lyons et al., 2011), and the parent strain used in the literature is the hypermotile *E. coli* K-12 wild-type MG1655 strain (Fraebel et al., 2017; Liu et al., 2019). Due to the IS insertion at the upstream of the *flhDC* promoter in the MG1655 strain, FlhDC was highly expressed (Supplementary Figure S7), activating the expression of the flagella to a nearly saturated level and inducing the hypermotile phenotype. Consequently, these mutated ClpXP protease-mediated accumulation of the FlhDC complex only enhanced the motility slightly in these hypermotile MG1655 strains. In contrast, since our parent strain is weakly motile (Figure 1B) and the expression level of FlhDC is low (Figure 1F), the effect of FlhDC accumulation mediated by the mutated ClpXP turns to be big. Thus, the effect of single-point mutations in ClpX on bacterial motility seems to depend on the background. We noted that none of the hypermotile mutations identified in this study belonged to the type of IS insertion at the upstream of *flhDC*. Given that there is already an insertion of Tn1000 existing at the upstream of *flhDC* in NCM3722 strains (Lyons et al., 2011), transposition of IS elements to the upstream of *flhDC* in NCM3722 strains might not occur as frequently as that found in MG1655 strains during the evolution on soft agar plates. Accordingly, this might increase the chances to isolate more various hypermotile mutants, for instance, the *clpX* mutants as identified here.

Taken together, we found three types of spontaneous mutations in ClpX, including V78F, Q289*, and L317R. The effect of Q289* and L317R mutations on the function of ClpX could be predicated due to the big chemical difference of the amino acids before and after mutation, whereas the effect of the mutated V78F to ClpX could be a mystery given that both amino acids are hydrophobic. We found that the mutated ClpX^V78F^P protease lost most of its ability to degrade the FlhDC complex (Figure 3C). Structure modeling showed that the main skeleton structures of the mutated ClpX^V78F^ and the wild-type ClpX were almost identical with a C_α_RMSD of 0.613 Å (Figures 4A–D), but the bulky aromatic side chain of F78 protruded into the ATP-binding pocket leaving much less space to accommodate the ATP (Figure 4D). Therefore, in the mutated ClpX^V78F^, the F78 might block ATP binding *via* the steric hindrance effect, resulting in the inactivation of the proteolytic activity of ClpXP. This hypothesis was further supported by our finding that the mutated ClpXP lost most of its ability to degrade FlhD *in vitro*, especially when ATP was deprived (Figures 4E–H). The *in vitro* study of ClpX has shown the importance of V78 and I79 to its ATP-dependent proteolytic activity (Stinson et al., 2013). To achieve appropriate binding affinity between ClpX and ATP, the contacts between ATP and the side chains of V78 and I79 play a key role (Stinson et al., 2013). In contrast, our study indicates that when the amino acid solely at position 78 is mutated to a residue with a larger side chain, the ATP hydrolyzation by ClpX was also largely affected (Figures 4E–H), which is consistent with the steric hindrance effect found in structure modeling (Figures 4A–D). This result further indicates the key role of V78 in controlling the activity of ClpX. Our proteomic results also supported the inability of ClpX^V78F^ to degrade known ClpXP targets other than FlhDC (Figure 5A). Hence, our findings supplied both the *in vivo* and *in vitro* proof and shed new lights on the importance of a proper size of ATP-binding pocket to the proteolytic activity of ClpXP. However, we surprisingly found that the effect of ClpX^V78F^ on the targets was not as general as expected. It appeared that the stability of certain proteins including FlhDC, RseA, RecN, MoaA, and ZapC was more responsive than the others, such as RpoS and YbaQ, to the ClpX^V78F^ mutation. This indicates that *in vivo*, different targets can be regulated by ClpXP to various extents, and it remains unclear whether there is a mechanism accounting for the specific effect of single-point mutations in ClpX on certain targets.

Furthermore, although FlhDC was known to be degraded by ClpXP, the signal tag of FlhDC recognized by ClpXP remains obscure. Since the signal tag of FlhD recognized by ClpXP has been identified here (RKKRA) and this tag sequence is highly similar to the known degradation signals of other proteins, this supports that FlhDC is directly degraded by ClpXP *in vivo* in a way similar to the other known targets of ClpXP.

Finally, although the hypermotile *E. coli* was identified to show growth cost (Wang and Wood, 2011; Ni et al., 2017) due to the energetic burden of the enhanced motility and flagella biosynthesis, possible bioprocesses that might be subject to this burden have never been explored. In this work, we identified that the motility genes together with biofilm formation and stress response genes showed antagonistic expression. In the hypermotile strains, in order to reallocate more resources for the biosynthesis of flagella, cells reduced the expression of genes related to biofilm formation and stress response accordingly (Figure 6B).

*E. coli* switches between the planktonic state and the biofilm state (Guttenplan and Kearns, 2013). Many studies reported the inverse expression of biofilm genes and motility genes when *E. coli* switches from the motile-planktonic state to the adhesive biofilm state (Domka et al., 2006; Domka et al., 2007; Hamilton et al., 2009). However, a few reported how the biofilm-related genes would change when *E. coli* switched oppositely or stayed in the planktonic state. Here, our result supports the logic that when *E. coli* chooses to stay in the planktonic state with high motility, inducing genes related to biofilm formation would challenge its choice, whereas reducing their expression would fit to its choice. Alternatively, during growth, *E. coli* is exposed to various stresses, including nutrient depletion (You et al., 2013; Gottesman, 2019), oxidative stress (Imlay, 2019), and acid stress (Kanjee and Houry, 2013; De Biase and Lund, 2015). Their successful growth and survival depend on the efficient adaptation to these stresses. Accordingly, the reduced expression of the stress-related genes identified in the hypermotile strains will weaken its fitness and survival (Figure 6D).

In conclusion, we identified that single-point mutations in ClpX boosted motility of *E. coli* largely *via* mediating the accumulation of the master regulator FlhDC. The gene of *clpX* seems to be another hot locus comparable to the upstream of the *flhDC* promoter region during the evolution of bacterial motility. At the molecular level, in the case of the mutant carrying ClpX^V78F^, the mutated V78F inactivated most of the protease activity of ClpXP *via* affecting ATP binding. And the signal tag of FlhDC recognized by ClpXP was also identified in this work. Importantly, the enhanced expression of motility genes paralleled with the reduced expression of genes functioning in stress resistance, which showed molecular reasons for the reduced fitness of the hypermotile bacteria. Taken together, our work highlights a highly efficient way *via* single-point mutation in *clpX* to boost the motility of bacteria.

## Materials and Methods

### Construction of Plasmids

To construct plasmid encoding the N-terminal His-tagged ClpX, the open reading frame (ORF) sequence of *E. coli* ClpX^WT^ or ClpX^V78F^ was PCR-amplified as a fragment flanked by the NdeI and HindIII sequences using primers of His-*clpX*-F (5′-CAT ATG ACA GAT AAA CGC AAA GAT GG-3′) and His-*clpX*-R (5′-AAG CTT GGT TAA TTA TTC ACC AGA TGC CTG-3′), and sub-cloned into plasmid pET28a. The resulted plasmid was designated as pCY519 (for ClpX^WT^) or pCY524 (for ClpX^V78F^).

To construct plasmid encoding the C-terminal His-tagged ClpP, the ORF sequence of *E. coli* ClpP was PCR-amplified as a fragment flanked by the NheI and XhoI sequences using primers of His-*clpP*-F (5′-GCT AGC TCA TAC AGC GGC GAA CGA GAT-3′) and His-*clpP*-R (5′-CTC GAG ATT ACG ATG GGT CAG AAT CGA-3′), and sub-cloned in plasmid pET24a. The resulted plasmid was designated as pCY526.

To construct plasmid encoding the N-terminal His-tagged FlhD, the ORF sequence of *E. coli* FlhD was PCR-amplified as a fragment flanked by the NdeI and HindIII sequences using primers of His-*flhD*-F (5′-CAT ATG CAT ACC TCC GAG TTG CTG AA-3′) and His-*flhD*-R (5′-AAG CTT TCA TGA TCA GGC CCT TTT CTT G-3′), and subcloned in plasmid pET28a. The resulted plasmid was designated as pCY531.

### Construction of *E. coli* Strains

The *E. coli* strains used and constructed in this study are listed in Supplementary Table S2. This mutated *fliC* of NCM3722 (Lyons et al., 2011) was complemented to the wild-type *fliC* of another widely used *E. coli* K-12 wild-type strain MG1655 *via* P1 transduction. In the genome, *fliC* locates between *fliA* and *fliDST* operon. To avoid the possible polar effect generated by adding a selection maker to *fliC*, we added a kanamycin marker to the downstream of *fliT*. In brief, the *kan* fragment encoding a kanamycin marker from the plasmid pKD4 was PCR-amplified and integrated into the chromosome right downstream of the *fliT* in MG1655 *via* the *λ* Red system (Datsenko and Wanner, 2000). The *fliT*-*kan* marker and *fliC* in MG1655 were transferred into NCM3722 *via* P1 transduction mediated by phage P1vir, resulting in CY598. The *fliC* in CY598 was confirmed to be the functioning wild-type *fliC* by DNA sequencing.

To introduce the point mutation of *clpX* ^G232T^ (ClpX^V78F^) into *E. coli* strains, the *clpX* ^V78F^-*cat* allele was first amplified by overlap PCR, using genomic DNA isolated from the hypermotile mutant CY706 and the plasmid pBSK-lcml (Li et al., 2019) as the template for *clpX* ^V78F^ and *cat*, respectively. Subsequently, the *clpX* ^V78F^-*cat* allele was integrated into the chromosome of target strains through the *λ* Red system (Datsenko and Wanner, 2000). Similarly, to complement the *clpX* ^V78F^ mutants, the *clpX* ^WT^-*cat* allele was introduced in target strains in the same manner as described above, except that genomic DNA isolated from CY598 was used as the template for *clpX* ^WT^. When necessary, the *cat* allele was removed by the Cre-LoxP system (Srividya et al., 2020).

To construct the ∆*clpX* mutant, the *cat* allele amplified from pBSK-lcml was introduced into target strains to replace the entire *clpX* gene in the chromosome, *via* the *λ* Red system (Datsenko and Wanner, 2000). When necessary, the *cat* allele was removed by the Cre-LoxP system (Srividya et al., 2020).

To construct a chromosomal fusion of C-terminal *clpX*-flag tag in *E. coli* strains, the *clpX*-*flag*-stop codon-*cat* allele was first obtained *via* overlap PCR amplification, using the plasmid pBSK-lcml as the template. Subsequently, the *clpX*-*flag*-stop codon-*cat* allele was integrated into the chromosome of target strains through the *λ* Red system (Datsenko and Wanner, 2000). The construction of C-terminal *flhC*-*flag* and *flhD*-tag-free-*cat* in *E. coli* strains was performed in the same manner as that described for *clpX*-*flag*.

To construct the ∆*clpP* mutant, the *cat* allele amplified from pBSK-lcml was introduced into target strains to replace the *clpP* gene in the chromosome, *via* the *λ* Red system (Datsenko and Wanner, 2000).

To construct the ∆*flhDC* mutant, the *cat* allele amplified from pBSK-lcml was introduced into target strains to replace the *flhDC* operon in the chromosome, *via* the *λ* Red system (Datsenko and Wanner, 2000).

### Growth Conditions

All *E. coli* strains were grown in the N^-^C^-^ minimal medium (You et al., 2013; Li et al., 2019) supplemented with 22 mM glucose (Sigma-Aldrich Corp., St. Louis, MO, United States), 20 mM NH_4_Cl (Sigma-Aldrich Corp.), and 0.2% (w/v) casamino acid (Aladdin, Shanghai, China), except where indicated. When needed, 100 μg/ml ampicillin (Aladdin), 50 μg/ml kanamycin (Sangon Biotech, Shanghai, China), or 25 μg/ml chloramphenicol (Solarbio, Beijing, China) was supplied in the medium. Except where indicated, all growths were carried out in a 37°C water bath shaker with aeration as described previously (Li et al., 2019).

### Isolation of Hypermotile Mutants

CY598 was grown to early exponential phase (OD_600_ = 0.3–0.4) and spotted on soft agar plates [0.5% beef extract, 1% peptone, 0.5% NaCl (Solarbio), and 0.4% agar (Sigma-Aldrich Corp.)]. The seeded soft agar plates were incubated at 37°C till swimming zones appeared. Subsequently, bacteria from several different areas of the swimming zones were streaked on regular LB plates [LB broth (Solarbio) and 1.5% agar (Sigma-Aldrich Corp.)] to isolate putative hypermotile mutant(s).

### Swimming Motility Assay on Soft Agar Plate

Early exponential phase cultures of strains were aliquoted and incubated at 37°C on soft agar plates [0.5% beef extract, 1% peptone, 0.5% NaCl (Solarbio), and 0.25% agar (Sigma-Aldrich Corp.)], and the diameters of swimming halos were measured.

### Time-Lapse Microscopy and Trajectory Analysis of Single Cell Motility

Bacterial strains were grown to early exponential phase (OD_600_ = 0.3–0.4) and diluted to an OD_600_ of approximately 0.002 with fresh culture medium in a six-well microtiter plate (polystyrene, tissue culture treated, flat bottom; Jet Bio-Filtration Co., Ltd., Guangzhou, China). A cover glass (18 square millimeters and 0.13–0.17 mm thick) was gently put on the top of the bottom surface of each well, providing a 15–20 micrometer (μm) deep channel. Movement of bacterial cells was observed using the 10 × phase-contrast objective of an inverted microscope (Nikon ECLIPSE Ti2) and videoed at 10.06 frames per second with a camera Nikon D5-Qi2. The trajectory of cells was analyzed using the software NIS-Elements AR (5.02.00). First, the video was cropped to limit the number of the analyzed cells, usually ranging from 30 to 80. Cell trajectories were captured *via* the program Cell Motility with the following settings: detect the targets as dark and round spots with various sizes; use 2.8–3.6 μm as the typical diameter for the targets; adjust the contrast ratio to subtract the background (eliminating debris or particles with a distinct density from the bacterial cells); check the box of “Add tracks”; check the box of “Delete trajectories with duration shorter than frames” and type 302 in the blank bar. Check the intact trajectory data listed after every run of the Cell Motility program, and if necessary, data for false (non-cell) targets could be further deleted manually. Finally, the darkness of the background could be adjusted by moving the LUTs bars, and trajectories were colored in a time-lapse manner for exported figures. To calculate the motility speed of a single cell, the same method was used as described above except for changing one of the settings in Cell Motility: check the box of “Delete trajectories with duration shorter than frames” and type 101 in the blank bar. Data of trajectories including path length (μm) and duration [second (s)] were exported, and the motility speed of a single cell was calculated as the path length divided by duration.

### Flagella Observation Under Transmission Electron Microscope

Strains were grown to early exponential phase (OD_600_ = 0.3–0.4) and harvested by centrifugation at 4,500 rpm. The pellets were washed with sterile double distilled water (DDW) and finally resuspended in DDW to an OD_600_ of approximately 1.0. 10 microliters (μl) of the resuspension described above was dropped on carbon-film-coated copper grids (400 mesh; Jingying Chemical Technology Co., Ltd., Guangzhou, China). After 15-min incubation at room temperature, fluid on the copper grids was absorbed with filter papers gently. Subsequently, 10 μl 0.6% (w/v) uranyl acetate was loaded on the copper grids to stain bacteria attached to the carbon film. After 1-min incubation at room temperature, fluid on the copper grids was absorbed with filter papers gently. Finally, copper grids with stained bacteria were completely dried at room temperature for at least 6 h. The flagella structure of bacterial strains on the copper grids was observed under transmission electron microscope HITACH HT7700.

### Quantitative Real-Time PCR

The total RNA of strains was extracted as previously described (Li et al., 2019). First, 1 μg of total RNA was reversed to cDNA by using PrimeScript™ RT reagent Kit with gDNA Eraser (Takara Bio Inc., Shiga, Japan), according to the manufacturer’s instructions. Subsequently, qRT-PCR was performed with TB Green Premix Ex Taq™ II (Tli RNaseH Plus; Takara Bio Inc.). In each reaction, the final concentration of primers was 0.5 micromolars (μM), and 100-time diluted cDNA was used as the template. Primers used for qRT-PCR are listed in Supplementary Table S3.

### Western Blots

30 ml of bacterial cells from the early exponential phase (OD_600_ = 0.3–0.4) were harvested by centrifugation at 4°C and subsequently resuspended in 300 μl PBS. The resuspension was subjected to sonication, followed by 20-min centrifugation at 4°C for collecting the supernatant. The protein concentration was determined using the Enhanced BCA Protein Assay Kit (Beyotime, Shanghai, China). For each strain, the same amount of protein (∼50 μg) was loaded for SDS-PAGE electrophoresis. To detect target proteins, transferred membranes were incubated with corresponding antibodies and followed by treatment with the SuperSignal™ West Pico PLUS Chemiluminescent Substrate (Thermo Fisher, Waltham, MA, United States). The membranes were exposed to Tanon-6100C (Tanon, Shanghai, China), and western blot bands were quantified using the software ImageJ. For each assay, RpoB was considered as a loading control. The primary antibodies used in this study are anti-FlhD (produced by Genscript, Nanjing, China), anti-RpoB (BioLegend, San Diego, CA, United States), and anti-Flag (Abcam, Cambridge, United Kingdom).

### Genome Sequencing

Genome sequencing of *E. coli* strains in this study was performed using the combination of Illumina and Pacbio techniques. Total DNA was isolated from the overnight LB culture of each strain. In brief, for Illumina sequencing, the qualified DNA samples were processed to construct a library according to the following steps. 1) The genomic DNA was treated into fragments. 2) The staggered ends of the fragments were repaired to blunt ends, using T4 DNA polymerase, Klenow DNA polymerase, and T4 PNK. 3) Specific adaptors were added to the 3′-ends of the fragments. Finally, the qualified libraries were subjected to cluster preparation and sequencing. For Pacbio sequencing, the qualified genomic DNA was broken into fragments >10 kb with g-TUBE. Damages and ends of the fragments were then repaired, followed by flanking both ends with a hairpin adapter, resulting in a structure known as SMRTbell. Primers were annealed to SMRTbell, which attached the fragments to the bottom of the ZWM polymerase, the enzyme performing the final sequencing. Clean data from the sequencing processes above were mapped to the NCM3722 genome (NCBI accession number for chromosome: CP011495.1, for plasmid F: CP011496.1) for reference assembly.

### Purification of Proteins

To purify the wild-type and mutated ClpX proteins, 60 ml bacterial cells of strain CY1222 or CY1223 harboring pCY519 or pCY524 were grown in LB broth (Solarbio) supplemented with 5 mM MgCl_2_ (Aladdin) and kanamycin at 37°C to an OD_600_ of ∼0.8. 0.2 mM IPTG (Solarbio) was subsequently added to the cell culture, and the cells were harvested after incubation at 12°C for 24 h. The pellets were washed twice using buffer A [50 mM Tris-HCl, pH 7.5 (Solarbio), 100 mM NaCl (Solarbio)] and resuspended in 1.5 ml buffer B [buffer A, 5 mM imidazole (Solarbio), 5 mM MgCl_2_]. The resuspended cells were subjected to a 40-min sonication program (every 5-s sonication was separated with a 5-s interval), and the supernatant was collected *via* 20-min centrifugation at 4°C. The supernatant was subsequently loaded to a gravity column containing Ni NTA Beads 6FF (Coolaber, Beijing, China), which had been balanced with buffer B in advance. The column was washed with buffer C (buffer A, 30 mM imidazole), and the proteins within the column were finally eluted using buffer D (buffer A, 250 mM imidazole). The elution containing ClpX was dialyzed against buffer E [buffer A, 10% glycerol (Macklin, Shanghai, China)].

To purify ClpP, 60 ml bacterial cells of strain CY1234 harboring pCY526 were grown in LB broth supplemented with kanamycin at 37°C to an OD_600_ of ∼0.8. 0.2 mM IPTG was subsequently added to the cell culture, and the cells were harvested *via* centrifugation after incubation at 30°C for 3 h. The pellets were washed twice using buffer A and resuspended in 1.5 ml buffer F (buffer A, 5 mM imidazole). The resuspended cells were subjected to a 40-min sonication program (every 5-s sonication was separated with a 5-s interval), and the supernatant was collected *via* 20-min centrifugation at 4°C. The supernatant was subsequently loaded to a gravity column containing TALON Metal Affinity Resin (Takara), which had been balanced with buffer F in advance. The column was washed with buffer C, and the proteins within the column were finally eluted using buffer D. The elution containing ClpP was dialyzed against buffer E.

To purify FlhD, 120 ml bacterial cells of strain CY1279 harboring pCY531 were grown in LB broth supplemented with kanamycin at 37°C to an OD_600_ of ∼0.8. 0.2 mM IPTG was subsequently added to the cell culture, and the cells were harvested *via* centrifugation after incubation at 30°C for 3 h. The same methods as described above for ClpP purification were used to obtain the FlhD protein, except that buffer G (buffer A, 60 mM imidazole) was used instead of buffer C during the wash step.

### *In Vitro* ClpXP Degradation for FlhD

The assay was conducted as previously described but with slight modifications (Takaya et al., 2012). In brief, a proper amount of ClpX, ClpP, and FlhD was incubated together at 37°C in the reaction buffer, comprising 50 mM HEPES-KOH, pH 7.6, 50 mM KCl, 10 mM Mg (CH_3_COO)_2_, and 5 mM DTT, at the presence of 0, 2.5, or 5 mM ATP. 10 μl of the mix for each reaction was aliquoted at time 0, 1, and 2 h and finally subjected to SDS-PAGE electrophoresis and Coomassie-brilliant-blue staining, for detecting the amount change of proteins. Protein bands were quantified using the software ImageJ.

### Transcriptome Study by RNAseq and Data Analysis

Bacterial cells from the early exponential phase (OD_600_ of ∼0.4) were used for RNAseq assays. MG1655 strains were grown in the N^-^C^-^ minimal medium (You et al., 2013; Li et al., 2019) supplemented with 22 mM glucose (Sigma-Aldrich Corp.) and 20 mM NH_4_Cl (Sigma-Aldrich Corp.). RNAseq assays were performed as previously described (Li et al., 2019). Three independent total RNA extraction and RNAseq assays were performed for strains derived from NCM3722 and two independent total RNA extraction and RNAseq assays were performed for strains derived from MG1655. RPKM method (Reads Per Kilo bases per Million Reads) (Mortazavi et al., 2008) was used to calculate the expression of each gene. Gene expression in the hypermotile mutants (CY706, CY708, CY1102) was individually compared to that in the weakly motile strains (CY598, CY1166). In total, four comparisons were performed, including gene expression in CY706 (resp. CY708 or CY1102) *versus* gene expression in CY598 and gene expression in CY708 *versus* gene expression in CY1166. Genes that showed a *p*-value ≤ 0.05 in each comparison by t-test and with the average fold change of the four comparisons higher than 1.5 were defined as differentially expressed genes (DEGs). Since only one comparison of the hypermotile and non-motile MG1655 strains was obtained, DEGs between the two transcriptomes of the MG1655 strains were characterized by edgeR (Robinson et al., 2010; Rajkumar et al., 2015) with 1.5-fold cutoff and adjusted *p*-value (*q*-value) < 0.01.

### Proteome Study and Data Analysis

300 ml of bacterial cells from the early exponential phase (OD_600_ = 0.3–0.4) were mixed with precool (from −80°C) 60% methanol (v/v) and harvested by centrifugation at 4°C. Cell pellets were washed with PBS, and the cells were collected again by centrifugation at 4°C. To perform proteomic analysis, the cells were first resuspended in four volumes of lysis buffer [8 molars urea (Sigma-Aldrich Corp.) and 1% protease inhibitor (Calbiochem, Merck, Darmstadt, Germany)]. The resuspension was subjected to sonication on ice using an ultrasonic processer (Scientz, Ningbo, China), followed by 10-min centrifugation at 4°C, to collect the supernatant and discard debris. The total protein concentration was determined by using the PCA kit. The total protein was subjected to 30-min reduction using 5 mM dithiothreitol (Sigma-Aldrich Corp.) at 56°C and subsequently incubated with 11 mM iodoacetamide (Sigma-Aldrich Corp.) at room temperature in darkness for 15 min. The protein solution was then diluted with 100 mM TEAB (Sigma-Aldrich Corp.), to alter the urea concentration to less than 2 molars. After the treatments above, the protein was digested overnight with trypsin (Promega, Madison, WI, United States) at a mass ratio of 1:50 (trypsin to protein) at 37°C, followed by 4-h digestion at a mass ratio of 1:100 (trypsin to protein).

The peptides from digestions above were dissolved in Solvent A [0.1% formic acid (Fluka, Sigma-Aldrich Corp.)] and separated by using the NanoElute UPLC system. In the process, the gradient of Solvent B [0.1% formic acid in 98% acetonitrile (Fisher Chemical, Thermo Fisher)] was set at the following series: Time 0–44 min, 6–22%; Time 44–54 min, 22–35%; Time 54–57 min, 35–80%; Time 57–60 min, 80%. This was conducted on the UPLC system at a flow-rate of 300 nanoliter/min. The separated peptides were ionized in a capillary ion source and subsequently analyzed by using timsTOF Pro mass spectrometry. The voltage used for the ion source was 1.6 kilovolt. The parent ion and its secondary fragments of the peptide were detected and analyzed by TOF. The scanning range of the secondary MS was set at 100–1,700 M/Z, and the data acquisition mode of Parallel Accumulation-Serial Fragmentation (PASEF) was used. After the collection of the primary mass spectrum, the secondary spectrum with the charge number of the parent ion in the range of 0–5 was collected in PASEF mode for 10 times. The dynamic exclusion time of tandem mass spectrometry scanning was set to 30 s to avoid the repeated scanning of the parent ion. Data from the mass spectra described above were searched using MaxQuant (v1.6.6.0) throughout the provided transcriptome database (Genome of NCM3722, NCBI accession number for chromosome: CP011495.1, for plasmid F: CP011496.1). In the meanwhile, the reverse decoy database was concatenated with the transcriptome database, to calculate FDR. In addition, databases of common contaminator peptides were used for analysis, to reduce the effect of contaminations. Several other key settings are listed here: Trypsin/P was set as the cleavage enzyme, and the number of missing cleavages was not allowed to be bigger than 2; the mass tolerance set for precursor ions in First search and Main search was both 40 ppm, and the mass tolerance set for fragment ions was 0.04 Da; the fixed modification was set as carbamidomethyl on Cys, and the variable modifications were set as oxidation on and acetylation on N-terminus of Met; the FDR for protein identification and modification identification was both set as 1%.

The expression of proteins in each sample was determined as LFQ intensity (MaxLFQ), which is based on the accurate quantification of peptides in MaxQuant, as previously described by Cox and colleagues (Cox et al., 2014). Two to three independent proteomic assays were performed for each strain. Protein expression in the strains carrying mutated or deleted ClpX (CY706, CY708, CY1102, CY1179) was individually compared to that in strains carrying wild-type ClpX (CY598, CY1166). In total, eight comparisons were performed. Proteins that showed a *p*-value ≤ 0.1 and with a fold change higher than 2 in at least one comparison were defined as differentially expressed proteins (DEPs). Then, DEPs identified by proteome assay were compared to DEGs identified by RNAseq, and DEPs not being identified as DEGs by the RNAseq assay were regarded as the target of the ClpXP protease.

### Survival Assay

Bacterial cells from the early exponential phase (OD_600_ of ∼0.4) were exposed to pH 2.5 for 5 min and plated on LB plates by series dilution. The survival rate was calculated by colony counting.

## Data Availability

The mass spectrometry proteomics data have been deposited to the ProteomeXchange Consortium *via* the PRIDE partner repository with the dataset identifier PXD023645. The whole genome data have been deposited to NCBI via the Genbank repository with the dataset identifier CP068279, CP068280, CP068281, CP068282, CP068283, and CP068284. Datasets of RNAseq have been deposited to NCBI via the GEO repository with the dataset identifier GSE165151 and GSE165438.
