# Supplementary material for: Gain of Spontaneous clpX Mutations Boosting Motility via Adaption to Environments in Escherichia coli
Source: Front Bioeng Biotechnol. 2021 Nov 24;9:772397. doi: 10.3389/fbioe.2021.772397 (PMC8652233; doi:10.3389/fbioe.2021.772397)
Supplement: Supplementary file 5 [file DataSheet1.PDF]

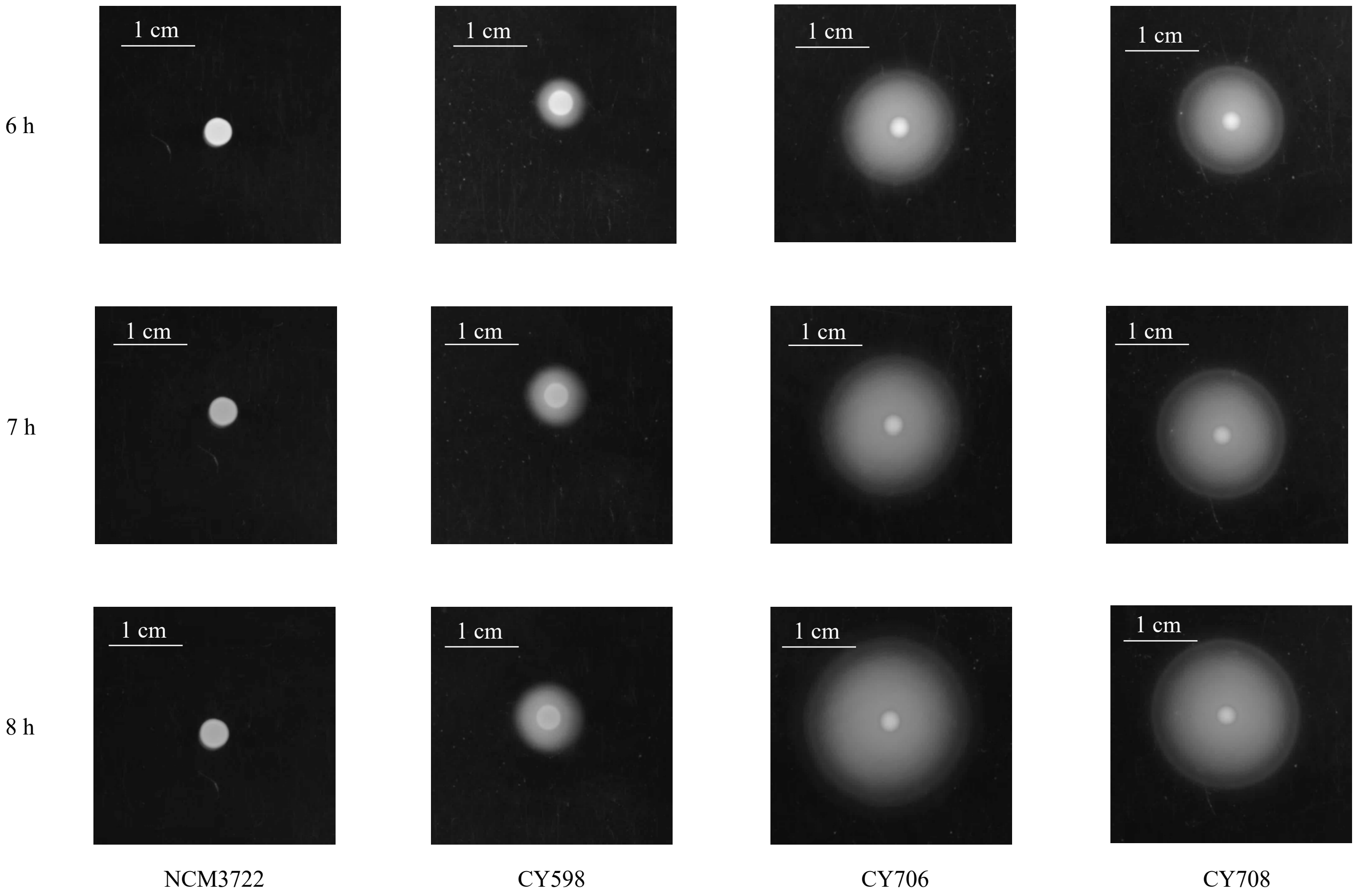

Figure S1. Swimming motility of NCM3722, CY598, CY706, and CY708 on soft agar plates (0.25%). Swimming halos were observed at indicated time points. One representative result of the three independent experiments is shown.

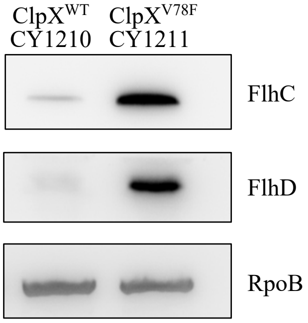

Figure S2. The protein levels of FlhC in strains carrying ClpX^{WT} or ClpX^{V78F}. CY1210 and CY1211 harboring the chromosomal *flhC*-flag fusion were respectively derived from CY598 (ClpX^{WT}) and CY1102 (ClpX^{V78F}). The protein levels of FlhD in these strains were also shown. RpoB was used as a loading control. One representative result of the three independent western blots is shown.

(A)

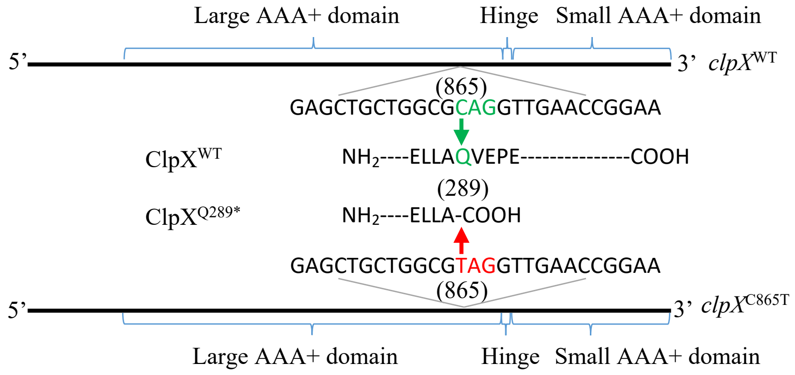

(B)

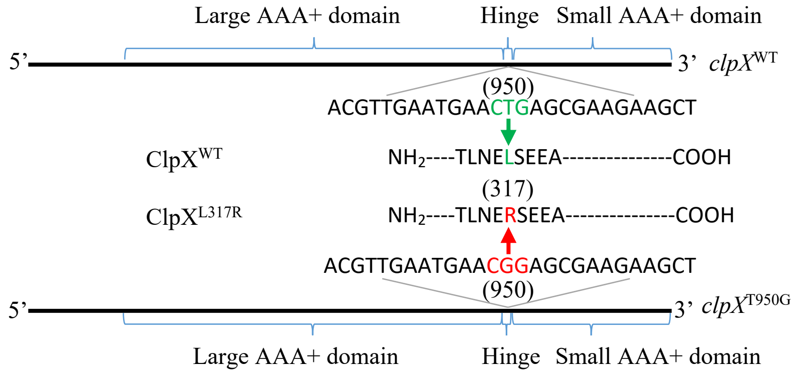

Figure S3. The DNA and amino acid sequences of more *clpX* variants identified. (A) The corresponding positions of the large AAA+ domain, small AAA+ domain and the hinge of the ClpX protein in its DNA sequence are marked. The C865T mutation in the *clpX* gene changed the codon of glutamine (CAG) into stop codon (UAG), resulting into $ClpX^{Q289*}$. (B) The T950G mutation in the *clpX* gene changed the codon of leucine (CUG) into the codon of arginine (CGG), resulting into $ClpX^{L317R}$.

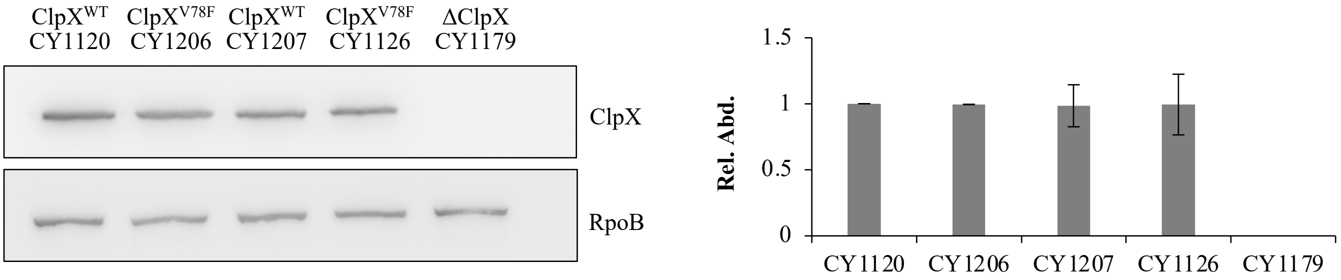

Figure S4. The protein levels of ClpX in strains carrying $ClpX^{WT}$ or $ClpX^{V78F}$. Strains harboring the chromosomal *clpX*-flag fusion were individually derived from CY598 ($ClpX^{WT}$) , CY708 ($ClpX^{V78F}$) , CY1166 ($ClpX^{WT}$) and CY1102 ($ClpX^{V78F}$). CY1179 carrying deleted ClpX was used as a negative control. RpoB was used as a loading control. One representative result of the western blots is shown (left plot). The protein level of ClpX in CY1120 was normalized to 1 and that in other strains were determined relative to this value. The relative protein level is shown as the average ± S.E.M. of the two independent experiments (right plot).

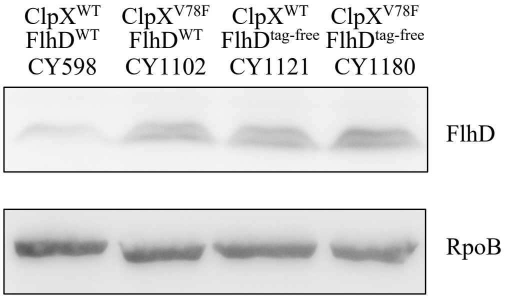

Figure S5. The protein levels of FlhD^{WT} or FlhD$^{tag\text{-}free}$ in strains carrying ClpX^{WT} or ClpX^{V78F}. Strains harboring the chromosomal FlhD$^{tag\text{-}free}$ was individually derived from CY598 (ClpX^{WT}) and CY1102 (ClpX^{V78F}). RpoB was used as a loading control. One representative result of three independent experiments is shown.

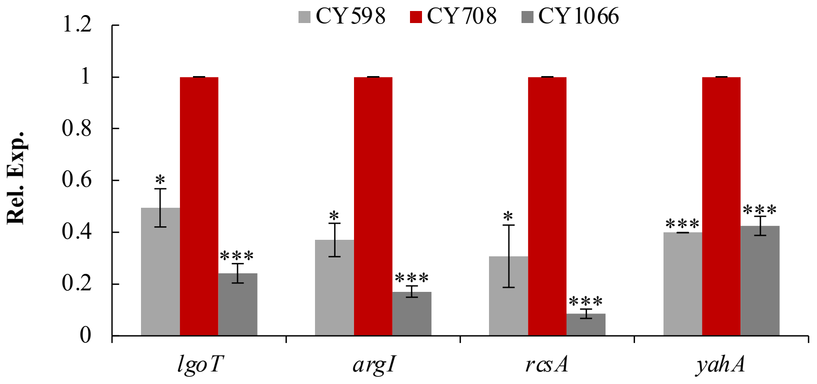

Figure S6. The expression of *lgoT*, *argI*, *rcsA*, and *yahA* in the three strains detected by qRT-PCR. CY1066 carrying deleted *flhDC* was derived from CY708. The mRNA level of each gene in CY708 was normalized to 1 and that in CY598 and CY1066 were determined relative to this value. The relative expression was shown as the average ± S.E.M. of three independent experiments. *, $p \leq 0.05$; ***, $p \leq 0.001$ versus the relative expression by CY708.

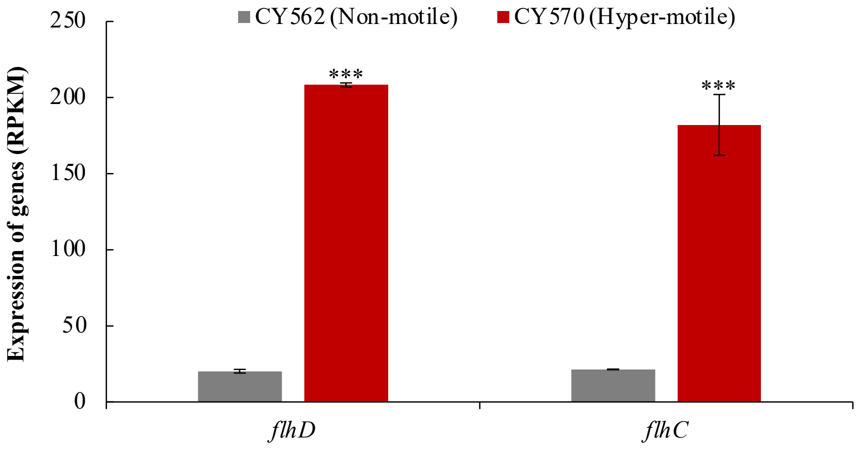

Figure S7. The expression of *flhDC* in the hyper-motile and non-motile MG1655 strains detected by RNAseq assays. The expression was shown as the average ± S.E.M. of two independent experiments. ***, $p \leq 0.001$ versus the expression of genes by CY562.

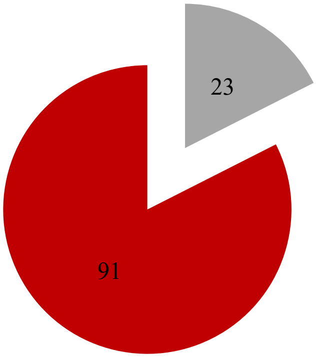

Figure S8. Pie chart showing the number of DEGs identified between the hyper-motile and non (weak)-motile strains derived from different backgrounds. 91 genes (red) were identified in strains derived from both the NCM3722 and MG1655 backgrounds and 23 genes (grey) were identified solely in strains derived from NCM3722.
